# A Generalized Form of Lichen Planus Pemphigoid Induced by an Oral Antidiabetic

**DOI:** 10.7759/cureus.31094

**Published:** 2022-11-04

**Authors:** Rokia Ajaaouani, Fouzia Hali, Farida Marnissi, Ahlam Meftah, Soumiya Chiheb

**Affiliations:** 1 Department of Venerology Dermatology, Ibn Rochd University Hospital, Casablanca, MAR; 2 Department of Venerology Dermatology, Ibn Rochd University Hospital Center, Hassan II University, Casablanca, MAR; 3 Department of Anatomical Pathology, Ibn Rochd University Hospital Center, Hassan II University, Casablanca, MAR; 4 Pharmacology, Ibn Rochd University Hospital Center, Casablanca, MAR

**Keywords:** sulphonylurea oral hypoglycaemic, gliclazide, induced, bullous skin disease, lichen planus pemphigoid

## Abstract

Lichen planus pemphigoid (LPP) is a rare autoimmune bullous dermatosis, although it is frequently idiopathic, the induced form is rare and there are few inducing drugs. We report a case of LPP induced by a gliclazide. A 68-year-old female patient with type 2 diabetes on gliclazide for three months presented with an eight-week history of generalized erythematous-papular eruption. Then she developed blisters on her forearms with oral mucosa involvement. A diagnosis of gliclazide-induced LPP was made based on a skin biopsy and imputability. The patient was treated with systemic corticosteroid with an improvement. LPP is a rare entity; its diagnosis is a challenge as it represents an overlap between lichen planus and bullous pemphigoid.

## Introduction

Lichen planus pemphigoid (LPP) is an uncommon acquired autoimmune bullous dermatosis marked by the coexistence of tense bullae and lichen planus lesions [1]. LPP is mostly idiopathic; however, it can be induced by certain medications, and some molecules have been implicated in induced LPP [2]. We report a case of pemphigoid lichen planus induced by an oral antidiabetic.

## Case presentation

**Figure 1:**
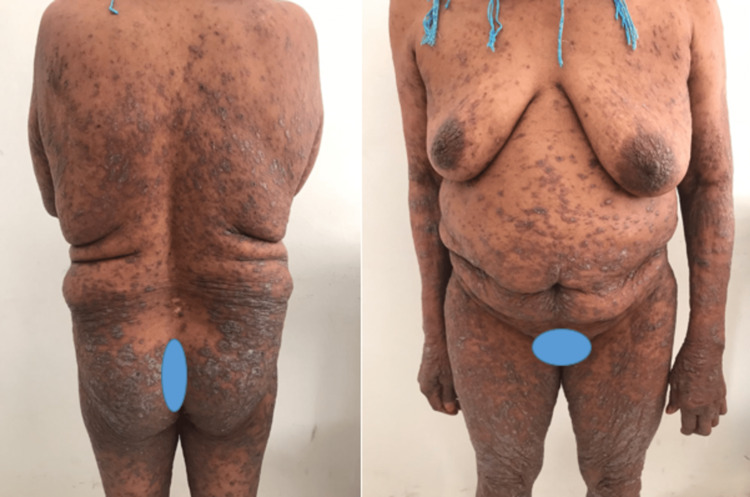
Generalized erythematous-papular eruption

**Figure 2:**
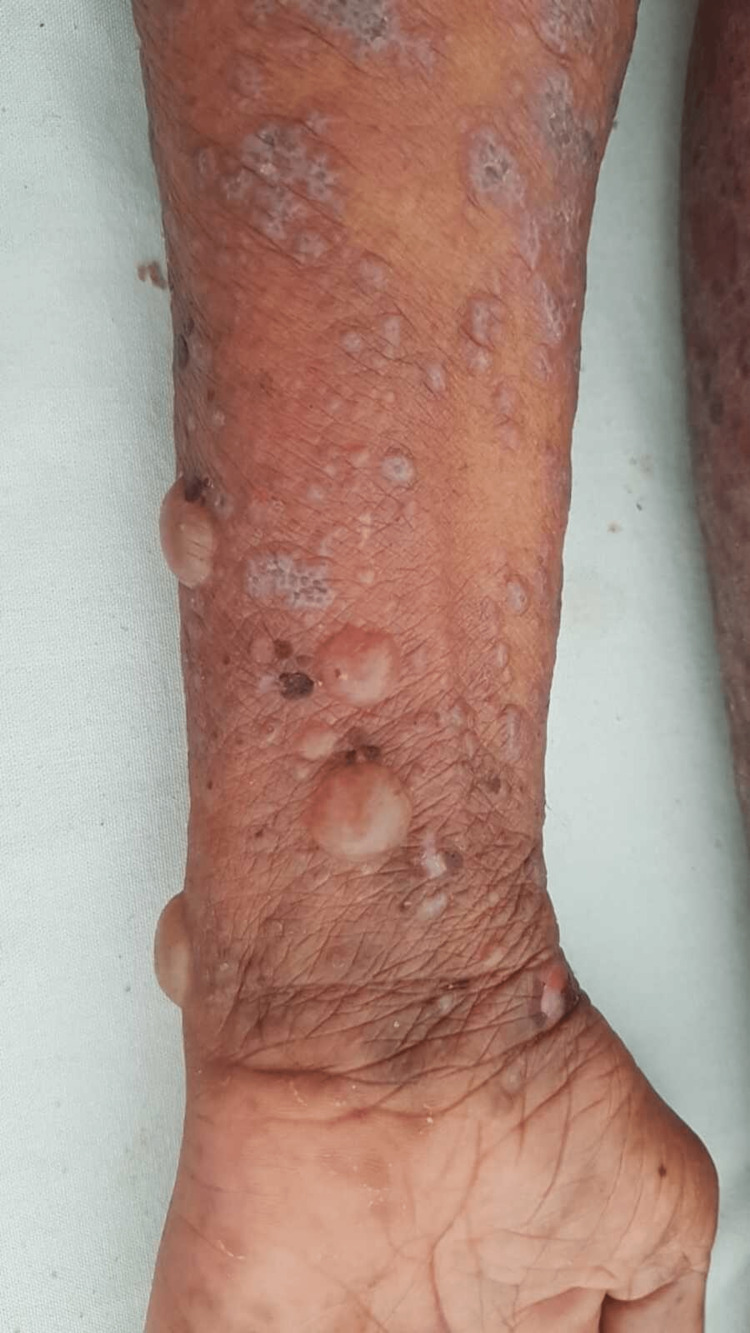
Violaceous papules topped by tense blisters

A 68-year-old female patient with type 2 diabetes on gliclazide for three months, without any other medication, developed pruritic papules on her trunk and extremities for two months, followed six weeks later by bullous lesions on her forearms. A clinical examination found generalized polygonal and violaceous, papules with Whickham streaks on the trunk, back, abdomen, legs, and thigh (Figure 1), topped by tense blisters (Figure 2).

**Figure 3:**
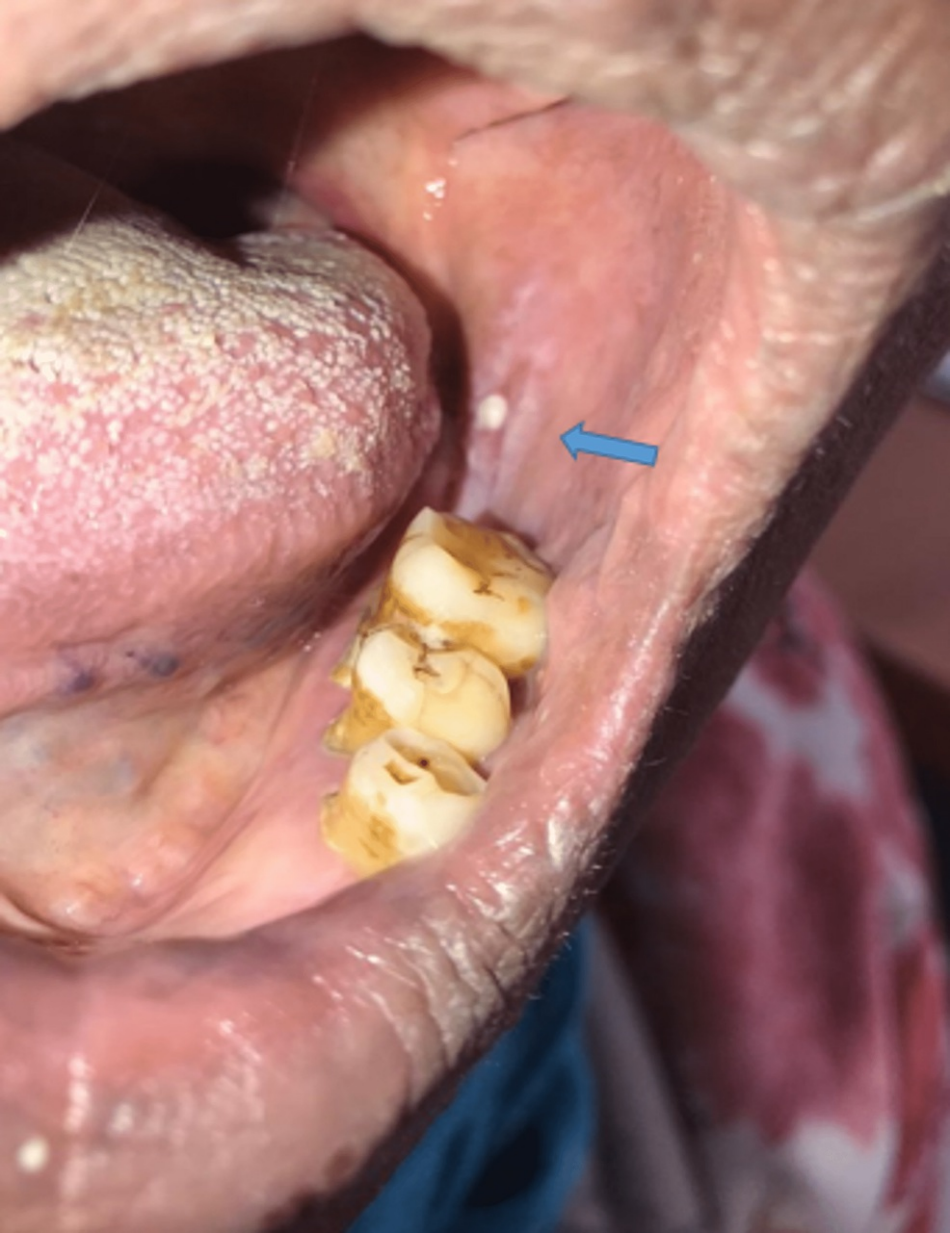
Whitish lichenoid network on the oral mucosa

Examination of the oral mucous found a whitish lichenoid network (Figure 3). A genital mucosa was intact.

Histopathological examination of the papule found an orthokeratotic epidermis, with exocytosis of neutrophils and apoptotic bodies. The dermis was the site of a lichenoid inflammatory infiltrate. The histology of the bullous found a subepidermal blister with inflammatory infiltrates made of eosinophil in the dermis. Direct immunofluorescence showed a junctional deposition of C3 and IgG. The blood count showed 780-element hyper eosinophilia. The pharmacovigilance survey found an accountability score according to the French I3B4 method. Gliclazide was discontinued and replaced with insulin; the patient received oral prednisone 0.5 mg/kg because of the extent of the lesions with improvement.

## Discussion

LPP is a rare and acquired autoimmune bullous dermatosis. It was first described by Kaposi [3] in 1982 as a typical case of lichen planus with a generalized bullous rash. It is diagnosed based on clinical, histopathological, and immunopathological features. Clinically, LPP is distinguished by two distinct primary skin lesions: lichenoid papules/plaques and tense blisters [1]. In LPP, lesions occur most commonly on the distal extremities but may present in a generalized form. Oral and conjunctival mucosal involvement has been reported and is more prevalent in women, mainly in the fourth or fifth decade of life [4].

The histopathology of a bullous lesion has the typical pattern of bullous pemphigoid (BP): subepidermal separation, with multiple eosinophils in the vesicular fluid and an eosinophilic infiltrate, while the histopathology of a lichenoid lesion has the typical characteristics of lichen planus [1]. The etiopathogenesis of LPP is not entirely clarified, with some authors considering it to be a variant of BP, and others a simple combination of lichen planus and BP [5]. LPP is uncommon and usually idiopathic; it is rarely associated with medication. The time to onset of the drug eruption ranged from 15 to 120 days. We report a case of LPP induced by gliclazide, given the clinical presentation: severe pruritus, extensive distribution of the cutaneous, mucosal presentation, and response to discontinuation of the drug; it is the cause of the current case of LPP, other drugs or therapies that have been implicated in the production of drug-induced LPP are listed in Table 1.

**Table 1:** Drug-induced of lichen plan pemphigoid ACE inhibitor: angiotensin-converting enzyme inhibitor, PUVA: psoralen plus ultraviolet-A radiation

Medications	Onset	Cutaneous distribution	Mucosa	Additionl treatment	references
ACE inhibitor					
Enalapril	6 years	Trunk, extremities	no	Topical steroid	[2]
Ramipril	3 weeks	Trunk, extremities	Yes	No	[7]
Captopril	2 weeks	Extremities	No	Topical steroid	[9]
Captopril	2 weeks	Trunk, extremities	No	Systemic steroid	[10]
Ramipril	4 weeks	Trunk, extremities	No	Systemic steroid	[11]
Cinnarizine	12 weeks	Lower extremities then trunk	No	Griseofulvin	[12]
PUVA	24 weeks	Trunk, extremities	No	Systemic and topical steroid	[13]
Simvastatin	4 weeks	Trunk, extremities	No	Topical steroid	[14]
Chinese herb	8 weeks	Trunk, extremities	Yes	Systemic steroid and dapsone	[15]
Weight reduction product	8 weeks	Trunk, extremities	No	Dapsone	[16]
hormone therapy	2 weeks	Trunk, extremities	No	Systemic steroid	[17]
vidagliptin	4 weeks	Abdomen, limbs	No	Systemic steroid, Mycophenolate mofetil	[5]

Pharmacovigilance databases are another precious tool to detect drugs that may cause the development of LPP. Gliclazide is a second-generation sulphonylurea oral hypoglycaemic drug, used widely in the treatment of type 2 diabetes in the population [6], including the elderly, because of its general safety and efficacy. A causal relationship is at least plausible when the LPP developed shortly after taking a new drug and completely resolved after stopping the drug suspected of triggering it [7]. In our case, the duration of the onset of the eruption was one month. Among the other skin side effects of gliclazide are itching, urticarial, rash, erythema, and flushing have also been reported in the literature [6]. Navarro-Triviño et al. [5] reported the first case of LPP induced by vidagliptin. From a pathophysiological point of view, Zillikens et al. [8] recognized a novel epitope within the NC16A domain of BP180, which appears to be uniquely recognized by sera from patients with LPP and is distinct from epitopes previously identified for classical BP. The epitope propagation theory is a plausible mechanism for drug-induced autoantibody formation. Firstly, by possible cytotoxicity, basal keratinocytes are damaged by the drug, resulting in exposure to BMZ antigens. Subsequent autoantibody formation leads to a bullous rash and BP-like immunofluorescence [7]. The management of drug-induced LPP is to stop the offending drug, change it to another therapeutic class and start systemic and/or topical corticosteroids depending on the extension and severity of the disease. In refractory cases, an immunosuppressant drug should be added (dapsone, azathioprine, MMF) [5].

## Conclusions

According to the literature, angiotensin-converting enzyme inhibitors are the most prone to LPP, while oral antidiabetics recognized by the lack of cutaneous side effects are increasingly inducing LPP. To our knowledge, this is the second case report of oral antidiabetic associated with the development of LPP. Gliptin is known to cause BP. Its pathophysiological mechanism and the report of our case may open the debate about whether LPP is really a variant of BP or if it should be considered a completely different entity.
